# Association of inflammatory biomarkers with physical and cognitive frailty in a Spanish population of older adults

**DOI:** 10.1007/s11357-025-01931-z

**Published:** 2025-10-16

**Authors:** Carlota Lema-Arranz, Ali Hemadeh, Natalia Fernández-Bertólez, Nuria Cibeira, Rocío López-López, Solange Costa, José Carlos Millán-Calenti, Laura Lorenzo-López, Vanessa Valdiglesias, Blanca Laffon

**Affiliations:** 1 Universidade da Coruña, Grupo DICOMOSA, CICA—Centro Interdisciplinar de Química e Bioloxía, Departamento de Psicología, A Coruña, Spain; 2 Instituto de Investigación Biomédica de A Coruña (INIBIC), Complexo Hospitalario, Universitario de A Coruña (CHUAC), Sergas, A Coruña, Spain; 3 Universidade da Coruña, Grupo NanoToxGen, CICA—Centro Interdisciplinar de Química e Bioloxía, Departamento de Biología, A Coruña, Spain; 4 Universidade da Coruña, Gerontology and Geriatrics Research Group, Instituto de Investigación Biomédica de A Coruña (INIBIC), Complexo Hospitalario Universitario de A Coruña (CHUAC), Sergas, A Coruña Spain; 5 EPIUnit—Instituto de Saúde Pública, Universidade do Porto, Porto, Portugal; 6 Environmental Health Department, National Institute of Health Doutor Ricardo Jorge, Porto, Portugal; 7 Laboratory for Integrative and Translational Research in Population Health (ITR), Porto, Portugal

**Keywords:** Physical frailty, Cognitive frailty, Inflammageing, Inflammatory biomarkers, Older adults

## Abstract

Frailty is a multifactorial geriatric syndrome characterized by increased vulnerability to stressors and associated with a higher risk of adverse health outcomes. Chronic low-grade inflammation has been proposed as a key pathophysiological mechanism underlying physical frailty, although its role in cognitive frailty remains undefined. In this cross-sectional study, we assessed the relationship between frailty status, both physical and cognitive, and plasma concentrations of six inflammatory biomarkers—C-reactive protein (CRP), interleukin 6 (IL-6), tumour necrosis factor alpha (TNF-α), soluble TNF-α receptor type II (sTNF-RII), high-temperature requirement serine protease A1 (HTRA1), and growth differentiation factor 15 (GDF15)—in a cohort of Spanish older adults (*N* = 150, ≥ 65 years old), classified according to Fried’s frailty phenotype and frailty index. The results showed notable differences between frailty phenotype and frailty index, and highlighted CRP, TNF-α, sTNF-RII, and GDF15 as key biomarkers significantly associated with physical frailty status, with CRP and TNF-α also discriminating pre-frail individuals. sTNF-RII stood out for its high predictive capacity, while GDF15 added value as an indicator of sustained cellular stress. Regarding cognitive frailty, CRP, TNF-α, and GDF15 displayed significant associations with this condition. sTNF-RII and HTRA1, scarcely studied in this context, showed promising and significant associations (specific for cognitive frailty in the case of HTRA1) that justify their inclusion in future research aimed at better understanding the inflammatory mechanisms involved in cognitive frailty.

**Graphical Abstract:**

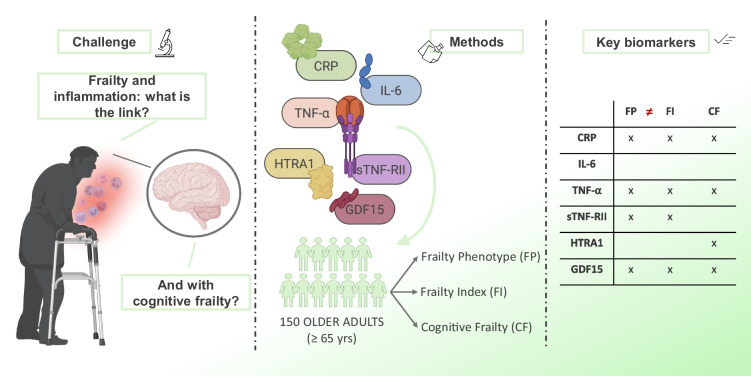

**Supplementary Information:**

The online version contains supplementary material available at 10.1007/s11357-025-01931-z.

## Introduction

The demographic pyramids have undergone notable changes in the last decades due to the ongoing process of population ageing [1]. Traditionally, this process has been attributed to the combination of declining birth rates and increased life expectancy [2], driven by advancements in medical, economic, and social fields. However, a recent study [3] confirmed that, while longevity has increased, the rate of ageing itself has remained constant, suggesting that individuals are living longer without a reduction in the speed of the biological deterioration associated with advancing age.

Given the heterogeneity in ageing manifestations, the term “frailty” was introduced as a more accurate indicator of biological ageing and survival than chronological ageing itself [4]. It is a complex multidimensional syndrome of loss of reserves in different physiological systems that determines a state of vulnerability associated with an increased risk of negative health outcomes [5, 6]. One of the most widely used tools to identify frailty is the “frailty phenotype”, proposed by Friet et al. [7], based on the presence or absence of five phenotypic criteria: muscle weakness, slow gait speed, unintentional weight loss, exhaustion, and low physical activity. Thus, frailty is considered a dynamic and bidirectional condition with three transitional stages (robustness, pre-frailty, and frailty) defined according to the number of positive criteria: none, 1 or 2, and 3 or more, respectively [7]. An alternative approach for frailty measurement, beyond purely physical characteristics, is the “frailty index” [8], based in the accumulation of different health deficits, including clinical signs, neurological examinations, psychological symptoms, and analytical laboratory parameters, among others. A higher number of health deficits, as indicated by an elevated frailty index, is linked to an increased risk of adverse health outcomes and mortality, especially when the index value exceeds 0.67.

Additionally, physical frailty is commonly associated with cognitive dysfunction in older people [9], likely due to shared underlying pathophysiological mechanisms. Consequently, the concept of “cognitive frailty” has been proposed as the co-occurrence of physical frailty and mild cognitive impairment (MCI), in the absence of overt dementia [9].

Frailty involves a multisystem dysregulation; however, the role of the immune system in age-related frailty is one of the most extensively documented [10–14]. Immunosenescence involves an age-related progressive decline in innate and adaptive immunity that contributes to increased susceptibility to infections, autoimmune diseases, cardiovascular diseases, cancer, etc. [10, 15]. Immunosenescence is closely associated with a low-grade chronic inflammation, a phenomenon known as “inflammageing”, which results from continuous antigenic stimulation [16]. This process is characterized by a sustained elevation in circulating levels of pro-inflammatory cytokines, along with acute-phase proteins like C-reactive protein (CRP), which impair the maintenance of immunological homeostasis [13, 17, 18], leading to a persistent pro-inflammatory state that disrupts multiple physiological systems, including the musculoskeletal, cardiovascular, and neuroendocrine systems [19, 20]. Inflammageing has been postulated as one of the main mechanisms involved in the pathophysiology of frailty [21, 22], and several recent systematic reviews and meta-analyses have evidenced the existence of a relationship between frailty and elevated levels of different inflammatory biomarkers, either restricting frailty identification to the Fried’s phenotype [23, 24] or using a variety of standard tools [25, 26]. Despite their potential, inflammatory molecules have not yet been systematically integrated into clinical frailty assessment, partly because, when considered in isolation, they cannot fully capture the complexity of the immunoinflammatory processes underlying this syndrome. This limitation highlights the need to identify and validate a broader panel of inflammatory biomarkers that can provide a more precise characterization of frailty status and clarify the role of the immune system in its pathophysiology.

Persistent systemic inflammation has been also associated with an increased risk of mental and cognitive disorders and neurodegenerative diseases [27]. Indeed, significantly elevated levels of inflammatory biomarkers have been consistently detected in individuals with depression [28] and Alzheimer’s disease [29]. However, the relationship between chronic inflammation and cognitive frailty remains unexplored, with just a few studies reporting elevated levels of inflammatory cytokines in individuals with cognitive frailty [30, 31].

This study aimed to analyse the association of biomarkers of inflammation with physical frailty, and to compare this association considering the phenotypic criteria [7] and the frailty index [8]. Moreover, it sought to provide insights into the relationship of MCI and cognitive frailty with inflammageing indicators, thereby contributing to a more comprehensive understanding of the pathophysiological mechanisms underlying those conditions. To this end, a cross-sectional study was conducted in a cohort of older adults (*N* = 155) aged 65 years and above and circulating levels of inflammatory mediators were quantified. In addition, since frailty is considered an intermediate status in the pathway of ageing from robustness to dependence [32], we explored the potential relationship between the inflammatory biomarkers and functional status in older adults.

## Methods

### Study subjects

In total, 150 individuals aged 65–96 (mean ± SD 73.32 ± 7.1; 67% women) were recruited from Galicia, North-western Spain. They were contacted through associations of older or retired people and day-care centres, and nursing homes. Ethical approval was granted by A Coruña—Ferrol Research Ethics Committee (reference number 2018/049). The study was conducted according to the Helsinki Declaration and International Conference of Harmonization guidelines. All participants signed a written informed consent. Sample size was calculated using G*Power (version 3.1.9.7) [33] considering an alpha level of *p* = 0.05, an effect size of *d* = 0.80 (large), and a power of 0.80. This calculation resulted in a group size of *n* = 21. Still, since different frailty classifications were used in the same population, different sizes were obtained for each of the three groups included in each classification.

Table 1 shows the general characteristics of the study population, classified according to the different frailty classifications (frailty phenotype, frailty index, and cognitive frailty). Participants underwent individual assessments conducted by interviewers with specialized training in clinical evaluation to ensure consistency in data collection. Each participant completed a structured questionnaire to obtain demographic, lifestyle, and medical information. The version of Montreal Cognitive Assessment (MoCA) questionnaire adapted and standardized by Ojeda et al. [34] for the Spanish population and adjusted for its sociodemographic characteristics was used to identify cognitive impairment. Nutritional status was evaluated by the Spanish version [35] of the Mini Nutritional Assessment-Short Form (MNA-SF) questionnaire [36], and comorbidity was estimated according to the Charlson index [37]. The functional status (i.e., the participants’ capacity to perform instrumental activities of daily living (IADL)) was evaluated using the Lawton-Brody IADL scale [38].

**Table 1:** Description of the study population

	Total	**Frailty phenotype**				**Frailty index**				**Cognitive frailty**			
		Healthy	Pre-frail	Frail	*p* value	Healthy	Pre-frail	Frail	*p* value	Healthy	MCI	Cognitive frail	*p* value
*No. individuals*	150	106	32	12		40	89	21		77	31	19	
*Age* *[mean* ± *SD (range)]*	73.5 ± 7.1 (65–96)	71.7 ± 5.6 (65–88)	74.1 ± 6.3 (65–89)	87.2 ± 6.1 (74–96)	< 0.001^c^	69.5 ± 4.1 (65–79)	73.0 ± 5.8 (6–88)	83.1 ± 8.4 (66–96)	< 0.001^c^	71.6 ± 5.7 (66–88)	71.7 ± 5.1 (65–79)	81.3 ± 9.9 (65–96)	< 0.001^c^
*Sex [N (%)]*					0.650^a^				0.349^a^				0.611^a^
*Male*	49 (32.7)	47 (34.9)	9 (28.1)	3 (25)		16 (40)	25 (28.1)	8 (38.1)		26 (33.8)	12 (38.7)	6 (31.6)	
*Female*	101 (67.3)	69 (65.1)	23 (71.9)	9 (75)		24 (60)	64 (71.9)	13 (61.9)		51 (66.2)	19 (61.3)	13 (68.4)	
*Smoking habit [N(%)]*					0.650^b^				0.716^b^				0.380^b^
*Non-smokers*	147 (98)	104 (98.1)	31 (96.9)	12 (100)		40 (100)	86 (96.6)	21 (100)		76 (98.7)	30 (96.8)	18 (94.7)	
*Smokers*	3 (2)	2 (1.9)	1 (3.1)	0 (0)		0	3 (3.4)	0		1 (1.3)	1 (3.2)	1 (5.3)	
*BMI* *[mean* ± *SD (range)]*	27.4 ± 4.0 (18.8–43.3)	27.4 ± 4.1 (18.8–43.4)	28.2 ± 4.2 (20.1–37)	25.7 ± 2.9 (21.5–30.7)	0.174^c^	25.3 ± 3.4 (18.8–34.4)	28.5 ± 4.1 (20.1–43.4)	27.3 ± 3.4 (21.5–34.1)	< 0.001^c^	27.6 ± 3.9 (18.8–43.37)	27.4 ± 4.3 (20.3–36)	26.5 ± 3.1 (21.5–33.25)	0.628^c^
*Nutritional Status [N (%)]*					0.006^a^				0.063^a^				0.004^b^
*Normal nutrition*	125 (83.3)	95 (89.6)	22 (68.8)	8 (66.7)		36 (90)	75 (84.3)	14 (66.7)		71 (92.2)	25 (80.6)	15 (78.9)	
*At risk or malnourished*	25 (16.7)	11 (10.4)	10 (31.3)	4 (33.3)		4 (10)	14 (15.7)	7 (17.3)		6 (7.8)	6 (19.4)	4 (21.1)	
*Alcohol consumption [N (%)]*					0.025^b^				0.035^a^				0.066^a^
*No consumption*	67 (44.7)	40 (37.7)	17 (53.1)	10 (83.3)		15 (37.5)	36 (40.4)	16 (76.2)		26 (33.8)	16 (51.6)	14 (73.7)	
*1–6 drinks/week*	46 (30.7)	37 (34.9)	7 (21.9)	2 (16.7)		15 (37.5)	28 (31.5)	3 (14.3)		30 (39.0)	7 (22.6)	2 (10.5)	
> *6 drink/week*	37 (24.7)	29 (27.4)	8 (25)	0 (0)		10 (25)	25 (28.1)	2 (9.5)		21 (27.3)	8 (25.8)	3 (15.8)	
*Tea/Coffee consumption [N (%)]*					0.158^b^				0.323^a^				0.132^a^
*0*	30 (20)	18 (17)	8 (25)	4 (33.3)		7 (17.5)	16 (18)	7 (33.3)		13 (16.9)	6 (19.4)	5 (26.3)	
*1–7 cups/week*	72 (48)	48 (45.3)	18 (56.3)	6 (50.0)		19 (45.5)	42 (47.2)	11 (52.4)		31 (40.3)	17 (54.8)	10 (52.6)	
< *7 cups/week*	48 (32)	40 (37.7)	6 (18.8)	2 (16.7)		14 (35)	31 (34.8)	3 (14.2)		33 (42.9)	8 (25.8)	4 (21.1)	
*Dietary supplements [N (%)]*					0.414^a^				0.029^a^				0.418^a^
*No*	90 (60)	64 (60.4)	17 (53.1)	9 (75.0)		31 (77.5)	47 (52.8)	12 (57.1)		45 (58.4)	21 (67.7)	13 (68.4)	
*Yes*	60 (40)	42 (39.6)	15 (46.9)	3 (25.0)		9 (22.5)	42 (47.2)	9 (42.9)		32 (41.6)	10 (32.3)	6 (31.6)	
*Living situation [N (%)]*					< 0.001^b^				< 0.001^b^				< 0.001^b^
*Community-dwelling*	138 (92)	106 (100)	31 (96.9)	1 (8.3)		40 (100)	88 (98.9)	10 (47.6)		77 (100)	31 (100)	9 (47.4)	
*Day care centre*	8 (5.3)	0 (0)	0 (0)	8 (66.7)		0 (0)	1 (1.1)	7 (33.4)		0 (0)	0 (0)	7 (35.0)	
*Nursing home*	4 (2.7)	0 (0)	1 (3.1)	3 (25.0)		0 (0)	0 (0)	4 (19.0)		0 (0)	0 (0)	3 (15.0)	
*Marital status [(N (%)]*					0.005^b^				< 0.001^b^				0.021^b^
*Single*	10 (6.7)	6 (5.7)	3 (9.4)	1 (8.3)		4 (10)	5 (5.6)	1 (4.8)		4 (5.2)	2 (6.5)	1 (5.3)	
*Married*	83 (55.3)	66 (62.3)	15 (46.9)	2 (16.7)		30 (75)	47 (52.8)	6 (28.6)		46 (59.7)	22 (71.0)	4 (21.1)	
*Widowed*	42 (28)	25 (23.6)	8 (25)	9 (75)		2 (5)	28 (31.5)	12 (57.1)		21 (27.2)	4 (12.9)	11 (57.9)	
*Divorced/separated*	15 (10)	9 (8.5)	6 (18.8)	0 (0)		4 (9.5)	99 (9.8)	2 (9.5)		6 (7.8)	3 (9.7)	3 (15.8)	
*Education (years) [N (%)]*					0.408^b^				0.001^a^				0.041^a^
< *9*	46 (30.7)	28 (26.4)	13 (40.6)	5 (41.7)		5 (12.5)	34 (38.2)	7 (33.3)		15 (19.5)	14 (45.2)	9 (47.4)	
*9–17*	72 (48)	52 (49.1)	14 (43.8)	6 (50)		18 (45)	43 (48.3)	11 (52.4)		43 (55.8)	9 (29)	8 (42.5)	
> *17*	32 (21.3)	26 (24.5)	5 (15.6)	1 (8.3)		17 (42.5)	12 (13.5)	3 (14.3)		19 (24.7)	8 (25.8)	2 (10.5)	
*Polypharmacy [N (%)]*					< 0.001^a^				< 0.001^a^				< 0.001^a^
*No*	117 (78)	93 (87.7)	20 (62.5)	4 (33.3)		49 (97.5)	72 (80.9)	6 (28.6)		69 (89.6)	26 (83.9)	9 (47.4)	
*Yes*	33 (22)	13 (12.3)	12 (37.5)	8 (66.7)		1 (2.5)	17 (19.1)	15 (71.4)		8 (10.4)	5 (16.1)	10 (52.6)	
*Comorbidity [N (%)]*					0.001^b^				< 0.001^a^				0.006^b^
*No*	128 (85.3)	96 (90.6)	26 (81.3)	6 (50.0)		37 (92.5)	80 (89.9)	11 (52.4)		70 (90.9)	28 (90.3)	11 (57.9)	
*Yes*	22 (14.7)	10 (9.4)	6 (18.8)	6 (50.0)		3 (7.5)	9 (10.1)	10 (47.6)		7 (9.1)	3 (9.7)	8 (42.1)	
*IADL dependence [N (%)]*					< 0.001^b^				< 0.001^b^				< 0.001^b^
*Independent*	80 (81.6)	63 (92.6)	16 (88.9)	1 (8.3)		24 (92.3)	51 (92.7)	5 (29.4)		42 (89.4)	22 (100)	4 (30.8)	
*Dependent*	18 (18.4)	5 (7.4)	2 (11.1)	11 (91.7)		2 (7.7)	4 (7.3)	12 (70.6)		5 (10.6)	0 (0)	9 (69.2)	
*Mild cognitive impairment (MoCA) [N (%)]*					< 0.001^b^				0.018^b^				< 0.001^b^
*No*	100 (66.6)	75 (70.8)	22 (68.8)	3 (25)		28 (70)	63 (70.8)	9 (42.8)		77 (100)	0 (0)	0 (0)	
*Yes*	50 (33.3)	31 (29.2)	10 (31.2)	9 (75)		12 (30)	26 (29.2)	12 (57.2)		0 (0)	32 (100)	20 (100)	

ANOVA analysis of variance, BMI body mass index, IADL independent activities of daily living, MoCA Montreal cognitive assessment

^a^ Chi-square test (bilateral)

^b^ Fisher exact test (bilateral)

^c^ ANOVA test (bilateral)

The inclusion criteria required individuals to be aged 65 or older, have normal or adequately corrected vision, and without a history of depression, degenerative neurological disorders, or dementia. Exclusion criteria included an inability to undergo assessment or refusal to provide informed consent. Additionally, participants were excluded if they were receiving medications classified under the Anatomical Therapeutic Chemical (ATC) category L (antineoplastic or immunomodulating agents) or had chronic infections, autoimmune diseases, or cancer, as these conditions are closely linked to immune system dysfunction and could introduce potential confounding effects.

### Frailty phenotype

The frailty phenotype status of each individual was determined based on the five criteria proposed by Fried et al. [7], using the standardized version to adapt the cutoff points to the Spanish population [39]. These criteria are based on the assessment of the presence or absence of the following phenotypic components:

(i) Unintentional weight loss, defined as a reduction of at least 4.5 kg over the past year, not attributable to dietary modifications or increased physical activity.
(ii) Self-reported exhaustion, assessed through two items from the Spanish-adapted version [40] of the modified 10-item Center for Epidemiological Studies-Depression (CES-D) scale [41].
(iii) Weakness, operationalized as grip strength in the lowest 20% at baseline, adjusted for gender and body mass index.
(iv) Slowness, defined as a walking speed in the lowest 20% at baseline, based on the time required to walk 15 feet (4.57 m), adjusted for gender and standing height.
(v) Low physical activity, determined as the lowest 20% at baseline based on a weighted score of weekly kilocalorie expenditure, calculated according to the Spanish validation [41] of the Minnesota Leisure Time Activity Questionnaire [42], and adjusted for gender.

Frailty was considered the presence of three or more of these criteria, pre-frailty as meeting one or two, and robustness as the absence of all five criteria.

### Frailty index

The frailty index was computed following the procedure described by Searle et al. [43] by assessing health deficits within the population, which encompassed chronic diseases, geriatric syndromes, functional limitations, nutritional deficiencies, and other pertinent factors. The index was calculated as the ratio of the cumulative number of health deficits present to the total number of deficits assessed [8]. A total of 27 health deficits were evaluated (Table S1). The resulting value (ranging from 0 to 1) was used for the classification of individuals into three groups: frailty index ≤ 0.08: non-frail; 0.09 < frailty index > 0.24: pre-frail; frailty index ≥ 0.25: frail [44].

### Cognitive frailty status

Cognitive frailty results from the simultaneous presence of both physical frailty and MCI, in the absence of severe neurocognitive impairment [9]. Accordingly, individuals were classified using the physical frailty phenotype criteria, alongside the MoCA questionnaire to determine the occurrence of cognitive frailty. MoCA evaluates domains such as memory, attention, language, executive functions, temporal and spatial orientation, and visuospatial ability. The maximum total score is 30 points, 26 being the cutoff score for cognitive impairment. Based on these criteria, individuals were categorized into three groups: healthy (no cognitive impairment and no positive frailty phenotype criteria), MCI (cognitive impairment with no positive frailty criteria), and cognitively frail (MCI plus 1–5 positive frailty phenotype criteria).

### Blood sample collection, storage, and analysis of immune biomarkers

Whole blood samples were obtained by venipuncture and collected into vacutainer tubes containing ethylenediaminetetraacetic acid (EDTA) between 9:30 and 10:30 h. Plasma was separated by centrifugation at 1000 × g for 10 min, after which the samples were aliquoted and stored at − 80 °C until analysis of inflammatory mediators. To ensure a “blind” study, all samples were coded at the time of collection.

Plasma levels of CRP, interleukin 6 (IL-6), tumour necrosis factor alpha (TNF-α), soluble TNF-α receptor type II (sTNF-RII), human high-temperature requirement serine protease A1 (HTRA1), and growth differentiation factor 15 (GDF15) were measured using quantitative sandwich enzyme-linked immunosorbent assays (ELISA) with commercial kits. All kits were sourced from R&D Systems, Inc. (Minneapolis, MN, USA), except for HTRA1, which was obtained from Cloud-Clone Corp. (CCC, Houston, TX, USA). Samples were diluted before analysis 50-fold for CRP and HTRA1, fourfold for GDF15, and tenfold for TNFRII. Spectrophotometric readings were conducted using a Spectrostar Nano microplate reader (BMG Labtech, Ortenberg, Germany) equipped with kinetic analysis software (Spectrostar Nano Control, BMG Labtech).

The precision of the assays, determined by intra- and inter-assay coefficients of variation (CV) as reported by the manufacturers, presented maximum values of 4.6% and 7% for CRP, 4.2% and 6.4% for IL-6, 3% and 8.4% for TNF-α, 4.8% and 5.1% for sTNF-RII, < 10% and < 12% for HTRA1, and 2.8% and 6% for GDF15.

### Statistical analysis

A general description of the population was carried out by univariate analysis based on frailty phenotype, frailty index, and cognitive frailty. Sociodemographic, clinical, and lifestyle characteristics were compared among the different groups using analysis of variance (ANOVA) for continuous variables and Chi-square test or Fisher exact test for categorical variables.

Preliminary univariate analyses were applied to assess the impact of physical and cognitive frailty, and of each one of the five phenotype criteria, on the inflammatory mediators. ANOVA followed by Tukey post hoc test or Student *t* test was applied to sTNF-RII, since these data followed a normal distribution (Kolmogorov–Smirnov goodness-of-fit test) after applying a logarithmic transformation. For the remaining inflammatory parameters (CRP, IL-6, TNF-α, HTRA1, GDF15), no significant improvement was observed with any transformation, so they were analysed using the Kruskal–Wallis and Mann–Whitney *U* tests, applying Bonferroni correction for multiple comparisons.

Multivariate linear regression models were conducted to assess the association between the different inflammatory biomarkers (CRP, IL-6, TNF-α, sTNF-RII, HTRA1, and GDF15) and physical frailty, cognitive frailty, and functional status (as the dependent variables). All models were performed using the log-transformed data and were adjusted for age, sex, and smoking status, and for parameter-specific actual confounders, including all that applied among the following: body mass index (BMI), nutritional status, alcohol, coffee, and dietary supplement consumption, living situation (community-dwelling, day-care centre or nursing home), marital status (single, married/partnered, widowed, separated/divorced), years of education, polypharmacy (use of more than five drugs per day), and comorbidity. For those inflammatory parameters significantly influenced by physical frailty, cognitive frailty and functional status new models were run introducing the biomarkers as dependent variables and including physical frailty and functional status or cognitive frailty and functional status mutually adjusted as independent variables, and adjusting also by age, sex, and smoking habit. All results are presented as mean ratios (MR) with 95% confidence intervals (95% CI).

To evaluate the discriminating capacity of the inflammatory biomarkers evaluated for the different frailty classifications, receiver-operating characteristic (ROC) curves were computed, considering as the standard the non-frail + pre-frail group in the case of frailty phenotype and frailty index, and the group composed of all non-cognitively frail individuals in the case of cognitive frailty. The Youden Index was derived to determine the optimal predictive value (OPV, cutoff point to discriminate physical or cognitive frailty), providing the best balance between sensitivity and specificity. Based on the OPV, a dichotomous variable was generated for each biomarker, classifying values as either above or below the determined threshold [45]. Subsequently, an ancillary analysis by logistic regression models was applied to estimate the odds of physical and cognitive frailty occurrence based on biomarker levels (as dichotomous variables). All models were adjusted by age, gender, smoking habit, and parameter-specific confounders.

Spearman rank correlation analysis was applied to estimate associations between variables. All analyses were performed with IBM SPSS software package V. 29 (SPSS, Inc.) and the STATA/SE software package V. 12.0 (StataCorp LP). Statistical significance was established at *p* value lower than 0.05.

## Results

The study population consisted of 150 older adults aged between 65 and 96 years (Table 1). Individuals classified as physically frail or cognitively frail were significantly older, with mean values over 80 years of age. There was a higher representation of females across all groups, exceeding 60% in all cases. No significant differences were found regarding smoking habits, with at least 94.7% of individuals in all groups being non-smokers. BMI only differed among groups classified according to the frailty index, with healthy individuals presenting lower values, and nutritional status differed among groups according to frailty phenotype and cognitive frailty, with a much lower proportion of individuals at risk of malnourishment or malnourished in the healthy groups. Absence of alcohol consumption was significantly more represented in the frail groups. No differences were observed regarding tea/coffee consumption, and the use of dietary supplements was significantly less frequent in healthy individuals classified according to the frailty index. The frequency of individuals attending daycare centres or living in nursing homes was significantly higher in the frail and cognitive frail groups. Significant differences were observed in marital status; among healthy, pre-frail, and MCI individuals, the majority were married/partnered, whereas the physically frail and cognitively frail groups were predominantly composed of widowed individuals. Significant differences were also observed in the number of years of education. For both frailty index and cognitive frailty, individuals in the frail and cognitively frail groups were less likely to have pursued education beyond 17 years, whereas this trend was reversed in the healthy groups. Regarding medication use, individuals classified as healthy, pre-frail, or MCI presented significantly lower rates of polypharmacy. As expected, comorbidity was significantly more prevalent in the groups of physical or cognitive frail individuals.

**Fig. 1:**
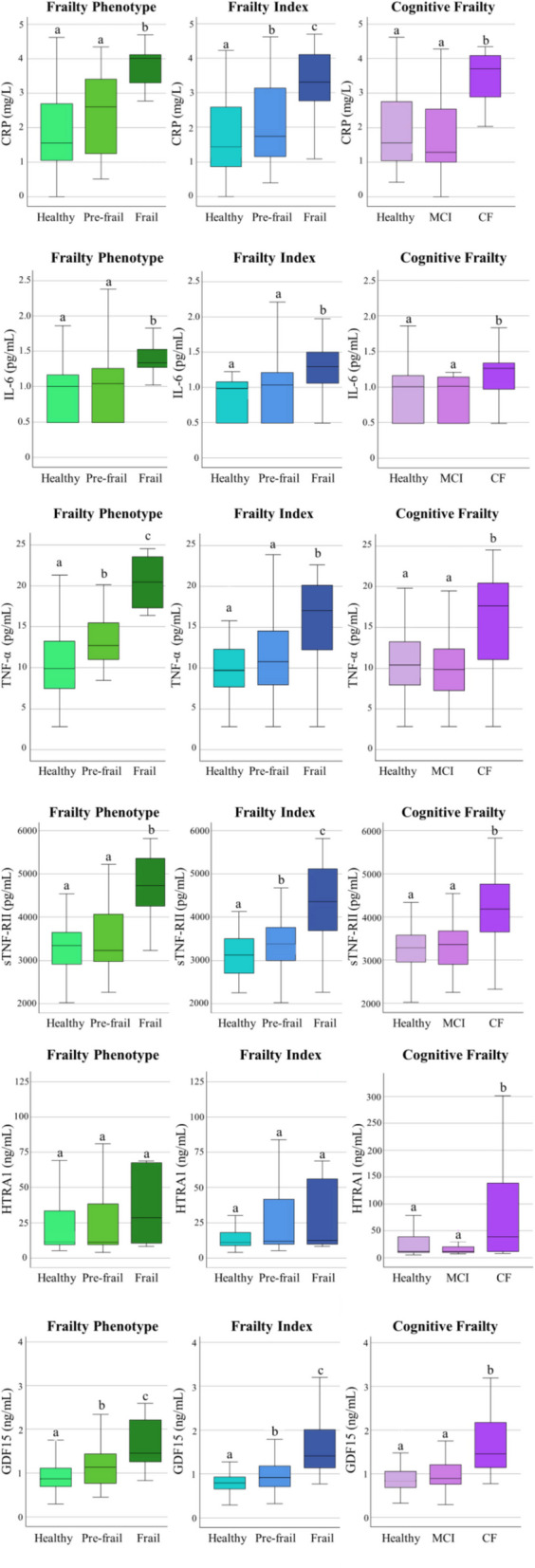
Results of inflammatory biomarkers in the study population, classified according to frailty phenotype, frailty index, and cognitive frailty (univariate analysis). Different letters indicate statistically significant differences between groups. Abbreviations: CRP C-reactive protein, IL-6 interleukin 6, TNF-α tumour necrosis factor alpha, sTNF-RII soluble TNF-α receptor II, HTRA1 high-temperature requirement serine protease A1, GDF15 growth differentiation factor 15, MCI mild cognitive impairment, CF cognitive frail

Figure 1 presents the values of inflammatory biomarkers for each group of older adults based on the different frailty classifications (frailty phenotype, frailty index, and cognitive frailty), with univariate analysis comparisons. All biomarkers exhibited significantly higher levels in both the frail and cognitive frail groups compared to the healthy group, except HTRA1, which only showed significantly increased levels in the cognitively frail individuals. Additional significance was observed for the progressive increase in the group of pre-frail individuals classified according to the frailty phenotype in TNF-α and GDF15, and classified according to the frailty index in CRP, sTNF-RII, and GDF15.

The results of the multivariate analysis of the biomarkers in relation to the frailty status are shown in Table 2. Supporting the univariate analysis, progressive increases in CRP levels were observed with increasing severity of physical frailty (according to frailty phenotype and frailty index), with 54% and 65% increases in the pre-frail group (significant only for the latter one), and 141% and 239% increases in the frail group compared to the healthy group, respectively. A significant 95% increase in CRP was also detected in the cognitively frail group. Similar results were observed for TNF-α, with significant differences between groups restricted to the frailty phenotype and in the cognitively frail group. In line with the univariate analysis, higher levels of sTNF-RII were obtained in the frail and cognitively frail groups, and significantly elevated levels of HTRA1 in the cognitively frail group compared to the healthy group (103% increase). Finally, statistically relevant increases in GDF15 were found in the frail group according to the frailty index, and in the cognitively frail group (32% and 35% increase, respectively). Influence of age, which was controlled for in all regression models, resulted significant for sTNF-RII and GDF15 in the three types of frailty classifications.

**Table 2:** Impact of frailty status (frailty phenotype, frailty index, and cognitive frailty) on inflammatory biomarkers (multivariate linear regression). Models adjusted for age, gender, smoking habit, and parameter-specific actual confounders

		Frailty phenotype			Frailty index			Cognitive frailty		
		Healthy	Pre-frail	Frail	Healthy	Pre-frail	Frail	Healthy	MCI	CF
CRP	MR	1	1.54	2.41^*^	1	1.65^*^	3.39^***^	1	0.65	1.95^*^
	95% CI		(0.96–2.46)	(1.03–5.60)		(1.06–2.55)	(1.63–7.06)		(0.41–1.04)	(1.03–3.66)
IL-6	MR	1	1.11	1.40	1	1.04	1.16	1	1.02	1.05
	95% CI		(0.84–1.49)	(0.83–2.34)		(0.79–1.37)	(0.73–1.82)		(0.76–1.38)	(0.71–1.55)
TNF-α	MR	1	1.35^*^	1.92^**^	1	1.03	1.18	1	1.02	1.57^**^
	95% CI		(1.06–1.71)	(1.25–2.94)		(0.81–1.30)	(0.76–1.92)		(0.79–1.30)	(1.13–2.19)
sTNF-RII	MR	1	1.04	1.22^**^	1	1.07	**1.18** ^**^	1	1.01	**1.11** ^*^
	95% CI		(0.97–1.12)	(1.07–1.39)		(0.99–1.14)	(1.04–1.34)		(0.94–1.09)	(1.00–1.23)
HTRA1	MR	1	1.13	1.44	1	1.44	0.88	1	0.79	**2.03** ^**^
	95% CI		(0.76–1.68)	(0.70–2.96)		(1.00–2.10)	(0.45–1.77)		(0.53–1.17)	(1.19–3.49)
GDF15	MR	1	1.16	1.16	1	1.13	**1.32** ^*^	1	1.09	**1.35** ^**^
	95% CI		(0.99–1.35)	(0.88–1.51)		(0.98–1.30)	(1.04–1.67)		(0.94–1.26)	(1.09–1.67)

Bold figures indicate statistically significant results

*MR* mean ratio, *CI* confidence interval, *CF* cognitive frail, *CRP* C-reactive protein, *IL-6* interleukin 6, *TNF-α* tumour necrosis factor alpha, *sTNF-RII* soluble TNF-α receptor II, *HTRA1* high-temperature requirement serine protease A1, *GDF15* growth differentiation factor 15

*p < 0.05, **p < 0.01, ***p < 0.001

Table 3 gathers the results of the analysis of the association between inflammatory biomarkers and functional status (IADL dependence). Significant increases were obtained for sTNF-RII and HTRA1 in the group of dependent individuals, with a notable twofold increase in the latter case.

**Table 3:** Effect of functional status (IADL dependence) on inflammatory biomarkers (multivariate linear regression). Models adjusted for age, gender, smoking habit, and parameter-specific actual confounders

		Independent	Dependent
CRP	MR	1	1.44
	95% CI		(0.94–2.19)
IL-6	MR	1	1.08
	95% CI		(0.62–1.92)
TNF-α	MR	1	1.45
	95% CI		(0.98–2.13)
sTNF-RII	MR	1	**1.16** ^*^
	95% CI		(1.02–1.31)
HTRA1	MR	1	**2.15** ^***^
	95% CI		(1.42–3.28)
GDF15	MR	1	1.14
	95% CI		(0.88–1.48)

Bold figures indicate statistically significant results

*IADL* instrumental activities of daily living, *MR* mean ratio, *CI* confidence interval, *CRP* C-reactive protein, *IL-6* interleukin 6, *TNF-α* tumour necrosis factor alpha, *sTNF-RII* soluble TNF-α receptor II, *HTRA1* high-temperature requirement serine protease A1, *GDF15* growth differentiation factor 15

*p < 0.05, ***p < 0.001

When functional status and physical frailty or cognitive frailty were mutually adjusted in the same model for those biomarkers that presented significance for both variables (sTNF-RII and HTRA1), the only model where both parameters remained significant was the one including cognitive frailty and functional status for HTRA1 (cognitive frail group MR 1.82, 95%CI 1.14–2.90, *p* = 0.012; IADL-dependent group MR 1.81, 95%CI 1.17–2.79, *p* = 0.008). Complete data are displayed in Table S2.

The analyses of the contribution of each frailty phenotype criterion to the variation of the inflammatory biomarkers studied (Fig. S1) showed that unintentional weight loss presented significant differences just for TNF-α, exhaustion for TNF-α, sTNF-RII, and GDF15, and all biomarkers were influenced by low physical activity, slow walking pace, and low grip strength, with the exception of HTRA1, which was only influenced by low grip strength (two-fold value in positive individuals than in negative ones).

**Fig. 2:**
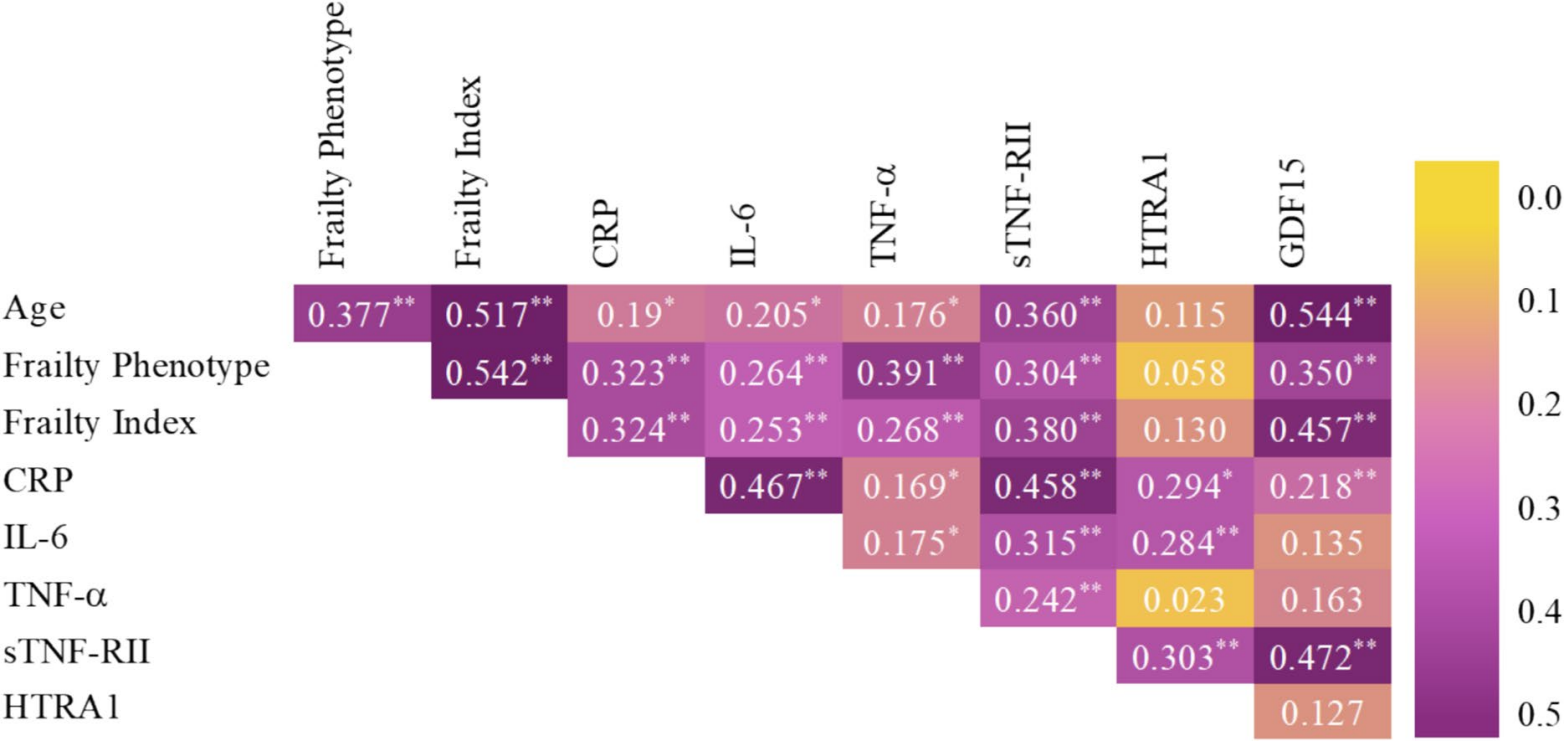
Spearman’s rank correlations between age, frailty phenotype (number of positive criteria), frailty index (score), and the inflammatory biomarkers. The heatmap displays correlation coefficients ranging from orange (absence of correlation) to violet (positive correlation) or to light yellow (negative correlation). Abbreviations: CRP C-reactive protein, IL-6 interleukin 6, TNF-α tumour necrosis factor alpha, sTNF-RII soluble TNF-α receptor II, HTRA1 high-temperature requirement serine protease A1, GDF15 growth differentiation factor 15. *p < 0.05, **p < 0.01, ***p < 0.001

The results from the correlation analysis between age, frailty phenotype (number of positive criteria), frailty index (score), and the inflammatory molecules are shown in Fig. 2. Age significantly correlated with both frailty measurements (*p* < 0.001) and all biomarkers, except HTRA1. Similarly, the two frailty estimations correlated significantly with all biomarkers (all *p* < 0.01), except HTRA1. Significant and notable correlations (*r* > 0.4) were observed for CRP and IL-6 and for sTNF-RII with CRP and with GDF15. Significant correlations with coefficient values between 0.4 and 0.2 were found between CRP and HTRA1, CRP and GDF15, IL-6 and sTNF-RII, IL-6 and HTRA1, TNF-α and sTNF-RII, and sTNF-RII and HTRA1. A weaker correlation was obtained for TNF-α with CRP and IL-6.

**Fig. 3:**
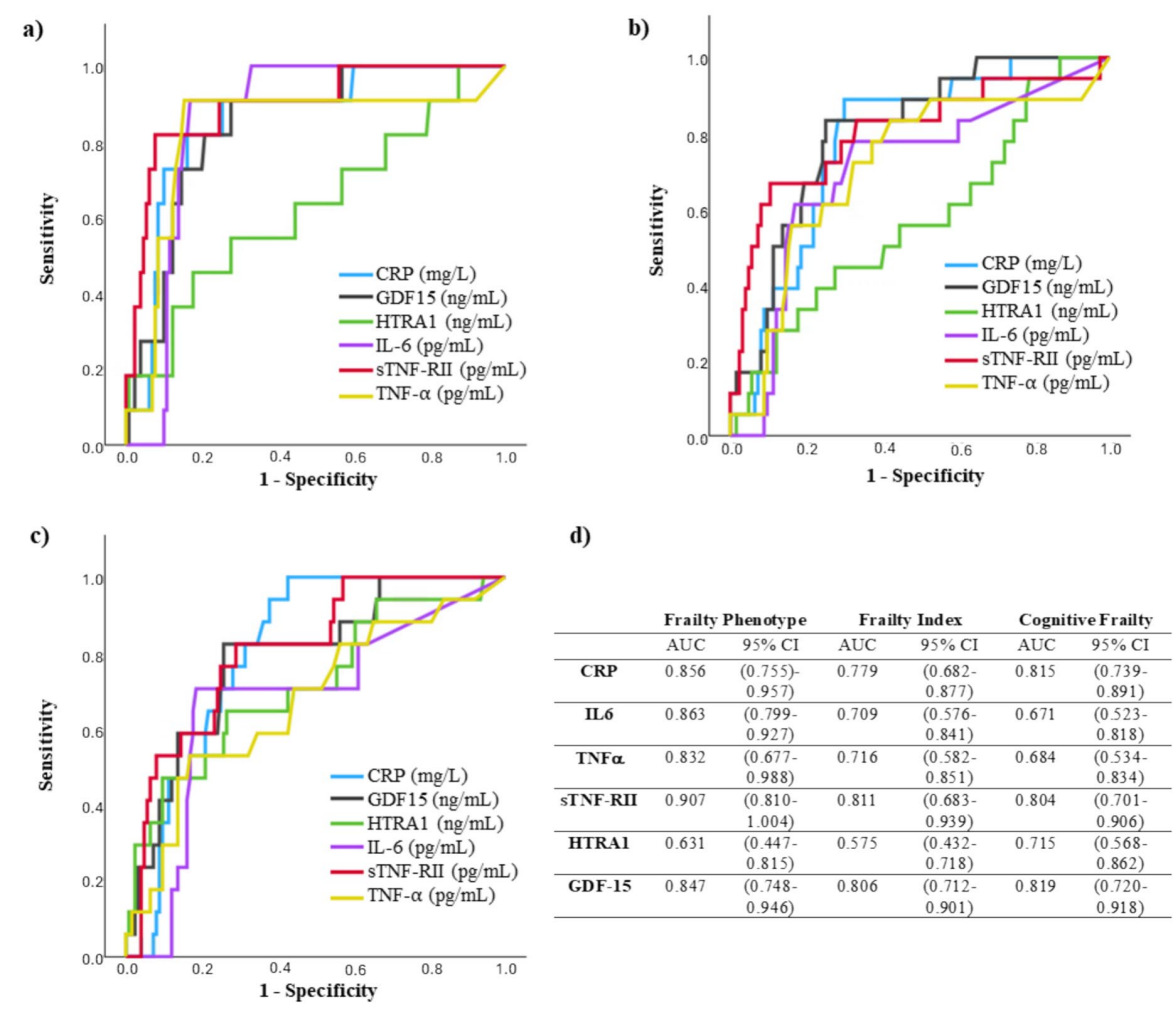
Receiver-operating characteristic (ROC) curves for the inflammatory biomarkers to predict frailty phenotype (**a**), frailty index (**b**), and cognitive frailty (**c**). Panel d shows the area under the curve (AUC) and the 95% confidence interval (95% CI) for each biomarker and frailty classification tool. CRP C-reactive protein, IL-6 interleukin 6, TNF-α tumour necrosis factor alpha, sTNF-RII soluble TNF-α receptor II, HTRA1 high-temperature requirement serine protease A1, GDF15 growth differentiation factor 15

ROC curves computed to assess the predictive value of inflammatory biomarkers for physical and cognitive frailty are shown in Fig. 3. In the case of frailty phenotype, values of the area under the curve (AUC) were always higher than 0.8, except for HTRA1, and exceeded 0.9 for sTNF-RII. AUC for frailty index exceeded 0.8 for sTNF-RII and GDF15, and 0.7 for CRP, IL-6, and TNF-α. Best values of AUC for cognitive frailty were observed for CRP, sTNF-RII, and GDF15 (higher than 0.8).

We also investigated the existence of differences between the presence or absence of frailty concerning the value of the immune biomarkers dichotomized according to their OPV for each frailty classification (Table 4). No biomarker was a significant independent predictor for physical frailty considering the frailty index. The analyses showed that levels of CRP and GDF15 over their OPV were significantly associated with an increased risk of frailty according to the frailty phenotype and cognitive frailty. Significantly higher levels of TNF-α and sTNF-RII were uniquely associated with an increased risk of frailty phenotype, while increased HTRA1 was associated with cognitive frailty (borderline significant, *p* = 0.058).

**Table 4:** Results of logistic regression analysis for inflammatory biomarkers based on physical and cognitive frailty. Biomarkers were dichotomized using their optimal predictive value (OPV) for each frailty classification. Adjustment for age, gender, smoking habit, and biomarker-specific actual confounders

Biomarker		OPV	No frailty (N)	Frailty (N)	OR	95%CI	***p*** value
Frailty phenotype	**CRP**	Below	88	26	1.00		
		Over	18	17	**3.48**	(1.33–9.29)	**0.012**
	**IL-6**	Below	88	24	1.00		
		Over	16	18	2.50	(0.92–6.08)	0.058
	**TNF-α**	Below	94	24	1.00		
		Over	12	19	**3.74**	(1.31–9.3)	**0.009**
	**sTNF-RII**	Below	102	27	1.00		
		Over	4	16	**8.75**	(1.85–20.25)	**0.003**
	**HTRA1**	Below	85	30	1.00		
		Over	19	11	1.06	(0.37–2.87)	0.905
	**GDF15**	Below	80	15	1.00		
		Over	24	26	**4.83**	(1.59–13.42)	**0.004**
Frailty index	**CRP**	Below	29	62	1.00		
		Over	11	47	1.76	(0.55–3.32)	0.239
	**IL-6**	Below	32	57	1.00		
		Over	8	49	2.34	(0.71–4.89)	0.105
	**TNF-α**	Below	28	51	1.00		
		Over	12	58	2.42	(0.90–5.12)	0.060
	**sTNF-RII**	Below	39	84	1.00		
		Over	1	25	6.32	(0.42–8.92)	0.122
	**HTRA1**	Below	31	70	1.00		
		Over	8	36	1.88	(0.88–6.71)	0.229
	**GDF15**	Below	33	62	1.00		
		Over	6	44	2.22	(0.44–5.11)	0.237
Cognitive frailty	**CRP**	Below	79	1	1.00		
		Over	51	18	**23.42**	(2.36–283.09)	**0.010**
	**IL-6**	Below	103	7	1.00		
		Over	24	12	2.84	(0.89–12.39)	0.143
	**TNF-α**	Below	110	8	1.00		
		Over	20	11	1.87	(0.29–7.32)	0.450
	**sTNF-RII**	Below	93	4	1.00		
		Over	37	15	3.92	(1.22–19.83)	0.063
	**HTRA1**	Below	93	7	1.00		
		Over	35	10	5.08	(1.27–34.3)	0.058
	**GDF15**	Below	93	2	1.00		
		Over	34	16	**13.32**	(1.59–46.88)	**0.013**

Bold figures indicate statistically significant results

*OR* odd*s* ratio, *CI* confidence interval, *CRP* C-reactive protein, *IL-6* interleukin 6, *TNF-α* tumour necrosis factor alpha, *sTNF-RII* soluble TNF-α receptor II, *HTRA1* high-temperature requirement serine protease A1, *GDF15* growth differentiation factor 15

## Discussion

There is a wide array of instruments for the detection of frailty in older adults, along with numerous modifications of the original validated scales. However, the most widely used methodologies for identifying physical frailty remain those proposed by Fried et al. [7], due to its simplicity and ease of implementation, and by Mitniski et al. [8], for its ability to capture the multidimensional nature of frailty through the accumulation of deficits. Still, the most appropriate method for identifying frailty remains unclear. Cesari et al. [46] suggested that these approaches should not be considered mutually exclusive, but rather complementary, with their application depending on the clinical status of the individual. In our study, group differences emerged depending on the classification method employed, with a larger proportion of individuals classified as robust by the Fried’s phenotype, and a majority classified as pre-frail using the frailty index, while both tools considered a minority of frail individuals. These discrepancies have been previously reported in the literature [47–49], reinforcing the need to develop frailty biomarkers that support frailty identification not solely based on physical and functional parameters, and that contribute to its early detection and to the development of clinically robust diagnostic tools and interventions before physical decline becomes evident.

Current evidence suggests that chronic inflammation is linked to cardiovascular disease, sarcopenia, osteoporosis, and increased risk of disability and mortality in older adults [50–53]. Inflammatory mediators trigger a cascade of immune-metabolic disruptions that may culminate in systemic functional decline. In addition, inflammageing has been demonstrated to play a central role in the pathogenesis of physical frailty [21, 22], justifying our focus on inflammatory biomarkers.

CRP, IL-6, and TNF-α are key mediators in the inflammatory cascade implicated in frailty. TNF-α, primarily secreted by activated macrophages, acts as an early pro-inflammatory cytokine that stimulates the production of other cytokines, including interleukin-1β (IL-1β) and IL-6 [54]. The latter one is produced by a variety of cells and plays a pivotal role in transitioning from acute to chronic inflammation inducing hepatic synthesis of acute-phase proteins such as CRP [55]. CRP, in turn, participates in opsonization and activation of the complement system, contributing to innate immune responses [56].

A systematic review and meta-analysis of 49 studies evaluated the role of inflammatory biomarkers in frailty phenotype among older adults [24]. The analyses provided evidence that elevated levels of CRP and IL-6 are consistently associated with increased frailty risk, reinforcing the central role of chronic low-grade inflammation in physical frailty pathogenesis. A weaker association was found for TNF-α. In addition, a recent systematic review of 44 studies on immune biomarkers and physical frailty, evaluated by a variety of standard tools, similarly found that CRP and IL-6 were consistently associated with frailty, with TNF-α being less strongly linked. Our current findings align with these results: all three biomarkers were significantly elevated in physically frail individuals in univariate analyses, although IL-6 lost significance after adjustment. Particularly strong associations were observed for CRP (increased by > 50% in pre-frail and > 140% in frail subjects compared to robust subjects, based on both frailty phenotype and frailty index) and TNF-α (35% increase in pre-frail and nearly twofold increase in frail individuals according to Fried’s phenotype), demonstrating their potential to discriminate pre-frail from robust individuals—a key capacity given the reversible nature of pre-frailty if detected early [57]. The ROC curve analyses demonstrated a high ability of CRP, IL-6, and TNF-α to predict physical frailty, with higher AUC values observed using frailty phenotype (0.86, 0.86, and 0.83, respectively) compared to the frailty index (0.78, 0.71, and 0.72, respectively). Additionally, logistic regression analyses confirmed that values of CRP and TNF-α over the OPV (3.38 mg/L and 16.22 pg/mL, respectively) are significantly related to the risk of physical frailty according to the frailty phenotype.

sTNF-RII is a type I transmembrane protein from the TNF receptor superfamily, primarily expressed by immune cells and mediating immune responses [58]. Its association with physical frailty has been explored in only three previous studies, which observed elevated levels in older individuals with frailty symptoms and a significant correlation with frailty status [59, 60], with one study [14] reporting an outstanding predictive capacity [61] for physical frailty (AUC 0.90). The present study confirms those previous reports, revealing a significant association between sTNF-RII levels and physical frailty status after adjustment. ROC curve analyses for sTNF-RII showed outstanding (AUC 0.91) and excellent (AUC 0.81) discriminant capacity [61] for frailty phenotype and frailty index, respectively, and logistic regression indicated that values above its OPV (4178.54 pg/mL) were significantly associated with frailty phenotype risk. All these results support its role as an independent factor in frailty pathogenesis. Notably, sTNF-RII exhibited the strongest correlations with other inflammatory biomarkers, suggesting it may play a key role in modulating the inflammatory processes associated with ageing.

HTRA1 is a heat stress-induced serine protease that combines proteolytic functions with chaperone activity. Its ability to negatively modulate the signalling of transforming growth factor beta (TGF-β), a fundamental cytokine in the modulation of the immune response due to its anti-inflammatory effect, has linked it to the regulation of inflammatory processes [62, 63]. In addition, elevated levels of this protein have been reported in several diseases, including sarcopenia, osteoarthritis, age-related macular degeneration, preeclampsia, and Alzheimer’s disease [63–66]. Nevertheless, investigation of HTRA1 as an inflammatory biomarker in frailty is limited to a single study [67], which found a significant association between plasma HTRA1 concentration and frailty status, suggesting its potential as an indicator of frailty progression. In contrast, our study did not find evidence supporting such association. Univariate and multivariate analyses, as well as ROC curves, failed to show significant differences or useful predictive capacity related to frailty status (as measured by both frailty phenotype and frailty index), suggesting its link to physical frailty might be less robust than previously proposed.

GDF15 is a pleiotropic cytokine from the TGF-β family, induced by cellular damage and metabolic stress, and is recognized as a sensitive marker of mitochondrial dysfunction and biological ageing [68–70]. It is upregulated in response to cellular stress or damage and can be induced by various growth factors and cytokines, including TGF-β, TNF-α, and IL-1β. Once expressed, GDF15 modulates the activity of multiple immune cell types [71]. GDF15 has been suggested as a convenient biomarker to identify older adults at risk of functional decline [72]. While studies reported elevated GDF15 levels in frailty and sarcopenia [73] and higher serum GDF15 concentrations associated with an increased risk of frailty both at baseline and after 2.2 years of follow-up [74], others did not find significant associations with frailty in older women, though a trend was observed in multivariate analysis [75]. These inconsistencies highlight the need for further research to clarify the role GDF15 as a frailty biomarker. In our univariate analyses, GDF15 levels differed significantly across all three groups (robust, pre-frail, frail) using both classifications for physical frailty, and multivariate analyses confirmed its ability to discriminate frail individuals. ROC curves indicated excellent predictive power for physical frailty (AUCs > 0.8) [61], and logistic regression results supported its potential as a frailty predictor. These findings underscore the relevance of GDF15 in identifying early stages of functional vulnerability and reinforce its diagnostic value in frailty. Our results suggest that GDF-15 may indicate a distinct pathophysiological profile, differing from that captured by classical inflammatory markers. Specifically, its lack of association with TNF-α and IL-6, key upstream mediators of CRP synthesis in hepatocytes [76], and its weak correlation with CRP imply that GDF15 is not primarily engaged in rapid immune responses. TNF-α, IL-6, and CRP are tightly connected to early immune activation and acute-phase responses [77], while GDF15 has been implicated in signalling pathways associated with prolonged mitochondrial stress, tissue injury, and chronic inflammation [78]. The strong correlation observed between GDF15 and sTNF-RII further supports this hypothesis, since sTNF-RII is considered a more stable surrogate of TNF-α activity [79] and has been associated with chronic inflammatory conditions, such as cardiovascular disease and metabolic syndrome [80]. This pattern suggests that GDF15 may serve as a more specific biomarker of sustained inflammation-related cellular damage, rather than acute systemic inflammation.

Given the growing body of evidence linking age-related neurodegeneration and associated disorders with physical frailty, the concept of cognitive frailty has emerged as a distinct and clinically relevant entity [9]. However, due to the nascent nature of this field, only four studies to date have investigated the relationship between inflammatory biomarkers and the presence of this syndrome [30, 31, 72, 81], despite evidence supporting its association with increased mortality risk [82].

In the study by Mu et al. [81], the authors examined levels of CRP, TNF-α, and IL-6 in individuals with cerebral small vessel disease and cognitive frailty. They reported a significant association between serum CRP and TNF-α levels and increased risk of cognitive frailty. Our findings are consistent with those results: both CRP and TNF-α were significantly higher in the presence of cognitive frailty (94% and 57% increase, respectively). Moreover, CRP demonstrated excellent discriminative performance in ROC curve analysis, with an AUC of 0.82. Logistic regression further confirmed that plasma CRP levels above 2.025 mg/L were associated with a markedly increased risk of cognitive frailty, highlighting its potential as a clinically useful biomarker. In line with Mu et al. [81], our results did not show a significant association between IL-6 and cognitive frailty either. However, a recent study by Diniz et al. [30] found significantly elevated IL-6 levels in older adults with cognitive frailty identified using the Mini-Mental State Examination (MMSE). It is important to note that the MMSE may lack sensitivity to detect cognitive decline associated with frailty, as no cognitive differences between pre-frail and non-frail groups were found using this test in previous studies [83]. In contrast, the MoCA was more effective in identifying subtle cognitive impairments related to frailty, highlighting its greater utility in this context.

Additionally, Kochlik et al. [72], investigated circulating levels of GDF15 in the context of cognitive frailty and depression in adults over 55 years of age, reporting significant associations for the simultaneous presence of both conditions. Our results align with their findings, as we observed elevated plasma GDF15 levels in individuals with cognitive frailty, and logistic regression identified a significantly increased risk of this condition in participants with levels exceeding 1.125 ng/mL.

To our knowledge, this is the first study to examine the potential association between plasma levels of the inflammatory proteins sTNF-RII and HTRA1 and cognitive frailty. Although sTNF-RII has previously been linked to cognitive decline [84–86], no prior studies have specifically explored its role in cognitive frailty. The present multivariate analysis revealed significantly elevated levels of this protein in individuals cognitively frail, and an excellent predictive capacity of sTNF-RII for cognitive frailty (AUC > 0.8 in the ROC curve).

While the discriminative value of HTRA1 for cognitive frailty, as reflected by an AUC of 0.72, was moderate (acceptable, according to [63]), logistic regression indicated a borderline significantly increased risk of cognitive frailty when plasma concentrations exceeded 22.90 ng/mL. In addition, significance for the difference in HTRA1 concentrations was only found in cognitive frailty, but not in physical frailty (neither frailty phenotype nor frailty index), both in the multivariate linear regression analysis and in the logistic regression analysis, indicating that HTRA1 could be a specific biomarker useful for the identification of cognitive frailty. Among the five phenotype criteria, HTRA1 was significantly higher just when low grip strength was present, and having MCI did not determine a higher HTRA1 concentration. Therefore, since cognitive frailty is defined by the simultaneous presence of frailty phenotype and MCI, it seems that the influence of cognitive frailty on HTRA1 is due to the concurrence of low grip strength (as the only component of physical frailty) and MCI (which do not modify HTRA1 by itself). Nevertheless, further research is warranted to confirm these findings and elucidate their potential clinical utility.

Differences in the inflammatory biomarkers regarding IADL dependence were only observed for sTNF-RII and HTRA1, with significantly higher concentrations in the group of dependent individuals. However, when frailty (physical or cognitive) and dependence were mutually adjusted in those cases where both showed significant differences, significance for both variables was maintained just in the case of cognitive frailty and functional status for HTRA1, indicating that both parameters have a strong influence on the obtained HTRA1 results. A role for HTRA1 in regulating muscle disease, strongly related to the functional capacity, has been suggested based on the upregulation of this protein in degenerating muscle, along with its capacity to alter the activation status of specific growth factors involved in controlling muscle growth [87].

As expected, both physical and cognitive frailty prevalence increased with age in our population. Indeed, the results of the correlation analyses showed that age correlated significantly with both scores of physical frailty. Besides, all inflammation biomarkers but HTRA1 correlated significantly with age, agreeing with the low-grade chronic inflammation during the ageing process (inflammageing). Therefore, the influence of frailty observed in this study for the inflammatory parameters could have been mediated by age. Still, correlation coefficients for the association of the biomarkers with age were in general weaker and with higher *p* values than those for their association with frailty scores. To control for the possible influence of age on the biomarkers when determining their association with frailty (physical or cognitive), all regression models applied included control for age. Significant influence of age was just detected for sTNF-RII and GDF15, in all models, suggesting that both frailty and age are important contributors for the variations in the concentrations of these particular biomarkers, but age does not significantly modify the concentrations of the other biomarkers, at least in the age range covered by this study (65–96 years).

Strengths of this study are: (i) the fact that frailty is not overrepresented in the sample since participants are community-dwelling individuals, they are not institutionalized; (ii) the comparison between frailty phenotype and frailty index, demonstrating that the two conceptual models do not lay exactly on the same physiological basis and supporting their complementarity; and (iii) the analysis of the association of inflammatory biomarkers with cognitive frailty, scarcely addressed so far and inexistent for some biomarkers, providing the grounds for future studies further exploring this subject. The study has also some limitations: (i) the cross-sectional design does not allow to determine the temporal sequence necessary to establish causality; and (ii) the size of the physical or cognitive frailty groups is small, preventing strong statistical evidence in the results.

## Conclusions

This study confirms the role of chronic inflammation in physical frailty, highlighting CRP, TNF-α, and sTNF-RII as key biomarkers significantly associated with frailty status. Additionally, CRP and TNF-α not only demonstrated strong discriminative ability for detecting frailty, but also effectively identified individuals in the pre-frail stage—a potentially reversible condition. sTNF-RII stood out for its high predictive capacity, while GDF15 added value as an indicator of sustained cellular stress. In contrast, HTRA1 showed no meaningful association with physical frailty. Notable differences were observed between frailty phenotype and frailty index, supporting the inherent dissimilarities between the two conceptual models.

Regarding cognitive frailty, CRP and TNF-α showed significant associations with this condition, reinforcing their clinical potential as detection biomarkers. GDF15 also demonstrated a consistent relationship, while sTNF-RII and HTRA1, scarcely studied in this context, showed promising and significant associations (specific for cognitive frailty in the case of HTRA1) that justify their inclusion in future research aimed at better understanding the inflammatory mechanisms involved in cognitive frailty.

## Supplementary Information

Below is the link to the electronic supplementary material.

ESM 1 Supplementary Material 1 (DOCX 1.02 MB)

## Data Availability

The anonymous data that support the findings of this study will be made available upon reasonable request through appropriate data sharing protocols.
