# Supplementary material for: Association of inflammatory biomarkers with physical and cognitive frailty in a Spanish population of older adults
Source: GeroScience. 2025 Oct 16;48(2):2063–84. doi: 10.1007/s11357-025-01931-z (PMC12972404; doi:10.1007/s11357-025-01931-z)

**SUPPLEMENTARY MATERIAL**

**Association of inflammatory biomarkers with physical and cognitive frailty in a Spanish population of older adults**

**Carlota Lema-Arranz · Ali Hemadeh · Natalia Fernández-Bertólez · Nuria Cibeira · Rocío López-López · Solange Costa · José Carlos Millán-Calenti · Laura Lorenzo-López · Vanessa Valdiglesias · Blanca Laffon**

C. Lema-Arranz · A. Hemadeh · B. Laffon

Universidade da Coruña, Grupo DICOMOSA, CICA—Centro Interdisciplinar de Química e Bioloxía, Departamento de Psicología, A Coruña, Spain

C. Lema-Arranz · A. Hemadeh · N. Fernández-Bertólez · V. Valdiglesias · B. Laffon

Instituto de Investigación Biomédica de A Coruña (INIBIC), Complexo Hospitalario Universitario de A Coruña (CHUAC), Sergas, A Coruña, Spain

N. Fernández-Bertólez · V. Valdiglesias

Universidade da Coruña, Grupo NanoToxGen, CICA—Centro Interdisciplinar de Química e Bioloxía, Departamento de Biología, A Coruña, Spain

N. Cibeira · R. López-López · J. C. Millán-Calenti · L. Lorenzo-López (*)

Universidade da Coruña, Gerontology and Geriatrics Research Group, Instituto de Investigación Biomédica de A Coruña (INIBIC), Complexo Hospitalario Universitario de A Coruña (CHUAC), Sergas, A Coruña, Spain

e-mail: [laura.lorenzo.lopez@udc.es](mailto:laura.lorenzo.lopez@udc.es)

S. Costa

EPIUnit—Instituto de Saúde Pública, Universidade do Porto, Porto, Portugal

Environmental Health Department, National Institute of Health Doutor Ricardo Jorge, Porto, Portugal

Laboratory for Integrative and Translational Research in Population Health (ITR), Porto, Portugal

| **Table S1** Health deficits and corresponding thresholds used for calculating the Frailty Index | | | |
| --- | --- | --- | --- |
| **Health Deficits Assessed** | **Assessment criteria or parameter cut-off points** | **Score** |  |
| Marital Status | Partnered or never married | 0 |  |
|  | Widowed or separated | 1 |  |
| Community Engagement | Engaged in social activities | 0 |  |
|  | Socially inactive | 1 |  |
| Activities of Daily Living | Fully independent | 0 |  |
|  | Mild impairment (1 task) | 0.25 |  |
|  | Moderate impairment (2 tasks) | 0.5 |  |
|  | Significant impairment (3-4 tasks) | 0.75 |  |
|  | Severe disability (5-6 task) | 1 |  |
| Instrumental Activities of Daily Living | No difficulty | 0 |  |
|  | Light difficulty (1-2 task) | 0.25 |  |
|  | Moderate limitations (3-4 tasks) | 0.5 |  |
|  | Advanced limitations (5-6 tasks) | 0.75 |  |
|  | Total dependency (7-8 tasks) | 1 |  |
| Walking speed (4m) | ≥0.8 m/s | 0 |  |
|  | <0.8 m/s | 1 |  |
| Handgrip Strength | Within expected range (normal) | 0 |  |
|  | Below normative threshold (low) | 1 |  |
| Cognitive Function (MMSE) | >24 points | 0 |  |
|  | 21-24 points | 0.25 |  |
|  | 18-20 points | 0.5 |  |
|  | 11-17 points | 0.75 |  |
|  | <10 points | 1 |  |
| Geriatric Depression Scale (GDS) | 0-2 points | 0 |  |
|  | 3-5 points | 0.25 |  |
|  | 6-8 points | 0.5 |  |
|  | 9-11 points | 0.75 |  |
|  | >11 points | 1 |  |
| Sedentarism | No | 0 |  |
|  | Yes | 1 |  |
| Body Mass Index (BMI) | 18.5-24.9 | 0 |  |
|  | Overweight (25-30) | 0.5 |  |
|  | Underweight or obese (<18.5 or >30) | 1 |  |
| Nutritional Evaluation (MNA) | 12-14 points | 0 |  |
|  | 8-11 points | 0.5 |  |
|  | 0-7 points | 1 |  |
| Unintentional Weight Loss (>5 kg last year) | No | 0 |  |
|  | Yes | 1 |  |
| Persistent Pain | Absent | 0 |  |
|  | Present | 1 |  |
| Ongoing Cancer or Active Treatments | Absent | 0 |  |
|  | Present | 1 |  |
| Cardiovascular conditions | Absent | 0 |  |
|  | Present | 1 |  |
| Chronic Respiratory Illness | Absent | 0 |  |
|  | Present | 1 |  |
| Hematological Diseases | Absent | 0 |  |
|  | Present | 1 |  |
| Kidney Diseases | Absent | 0 |  |
|  | Present | 1 |  |
| Neurological Disorders (Central System) | Absent | 0 |  |
|  | Present | 1 |  |
| Neurological Disorders (Peripheral) | Absent | 0 |  |
|  | Present | 1 |  |
| Gastrointestinal Issues | Absent | 0 |  |
|  | Present | 1 |  |
| ENT Disorders (Ear, Nose, Throat) | Absent | 0 |  |
|  | Present | 1 |  |
| Musculoskeletal Problems | Absent | 0 |  |
|  | Present | 1 |  |
| Mental Health Diagnosis | Absent | 0 |  |
|  | Present | 1 |  |
| Diabetes mellitus | No diagnosis | 0 |  |
|  | Diagnosed | 1 |  |
| Hormonal/Endocrine Conditions | Absent | 0 |  |
|  | Present | 1 |  |
| Polypharmacy (≥6 prescriptions) | No | 0 |  |
|  | Yes | 1 |  |

| **Table S2 Impact of frailty status (frailty phenotype, frailty index and cognitive frailty) and IADL dependence on sTNF-RII and HTRA1 inflammatory biomarkers (models mutually adjusted, and additionally adjusted by age, sex and smoking habit)** | | | | |
| --- | --- | --- | --- | --- |
| **sTNF-RII** | |  | **sTNF-RII** | |
|  | **MR (95%CI)** |  |  | **MR (95%CI)** |
| **Frailty phenotype** |  |  | **Frailty index** |  |
| ***Healthy*** | **1** |  | ***Healthy*** | **1** |
| ***Pre-frail*** | 1.09 (0.99–1.21) |  | ***Pre-frail*** | 1.02 (0.93–1.11) |
| ***Frail*** | **1.20**^*^(1.00– 1.43) |  | ***Frail*** | **1.18^*^** (1.02– 1.37) |
| **IADL dependence** |  |  | **IADL dependence** |  |
| ***Independent*** | **1** |  | ***Independent*** | **1** |
| ***Dependent*** | **1.08** (0.93-1.25) |  | ***Dependent*** | 1.09 (0.96–1.24) |
| **sTNF-RII** | |  | **HTRA1** | |
|  | **MR (95%CI)** |  |  | **MR (95%CI)** |
| **Cognitive Frailty** |  |  | **Cognitive Frailty** |  |
| ***Healthy*** | **1** |  | ***Healthy*** | **1** |
| ***MCI*** | 1.05 (0.96–1.15) |  | ***MCI*** | 1.07 (0.78– 1.47) |
| ***Cognitive frail*** | 1.10 (0.96–1.26) |  | ***Cognitive frail*** | **1.82^*^** (1.14–2.90) |
| **IADL dependence** |  |  | **IADL dependence** |  |
| ***Independent*** | **1** |  | ***Independent*** | **1** |
| ***Dependent*** | **1.14^*^** (1.00–1.29) |  | ***Dependent*** | **1.81^**^**(1.17–2.79) |
| MR, mean ratio; CI, confidence interval; sTNF-RII, soluble TNF-α receptor II; HTRA1, high-temperature requirement serine protease A1; IADL: Instrumental Activities of Daily Living. Bold figures indicate statistically significant results  *p<0.05, **p<0.01 | | | | |

**Fig. S1** Representation of the contribution of each frailty phenotype criterion (unintentional weight loss, exhaustion, low physical activity, slow walking pace, and low grip strength) to the variation in inflammatory biomarkers. Abbreviations: CRP, C-reactive protein; IL-6, interleukin 6; TNF-α, tumour necrosis factor Alpha; sTNF-RII, soluble TNF-α receptor II; HTRA1, high-temperature requirement serine protease A1; GDF15, growth differentiation factor 15. *p < 0.05, **p < 0.01, significant differences compared to the absence of the respective criterion


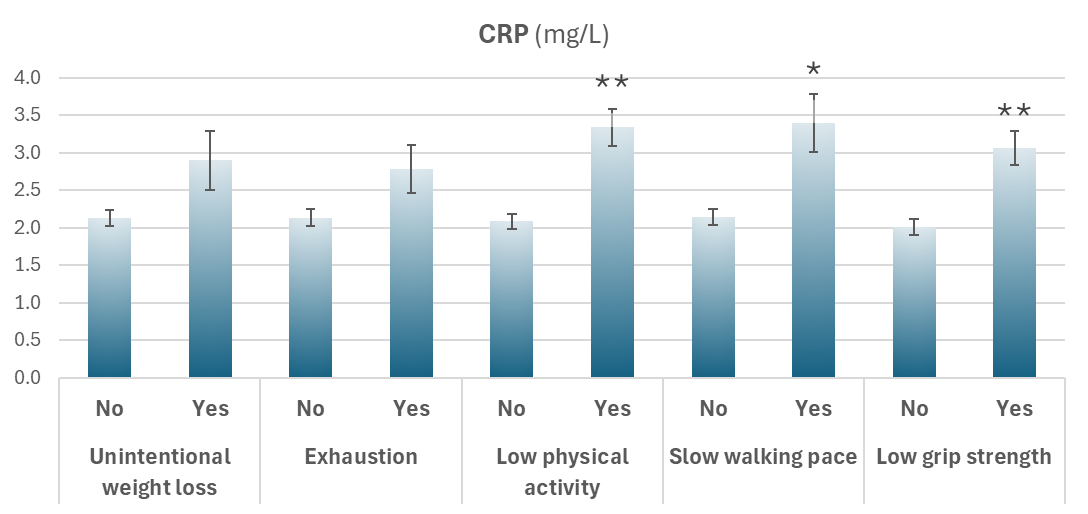

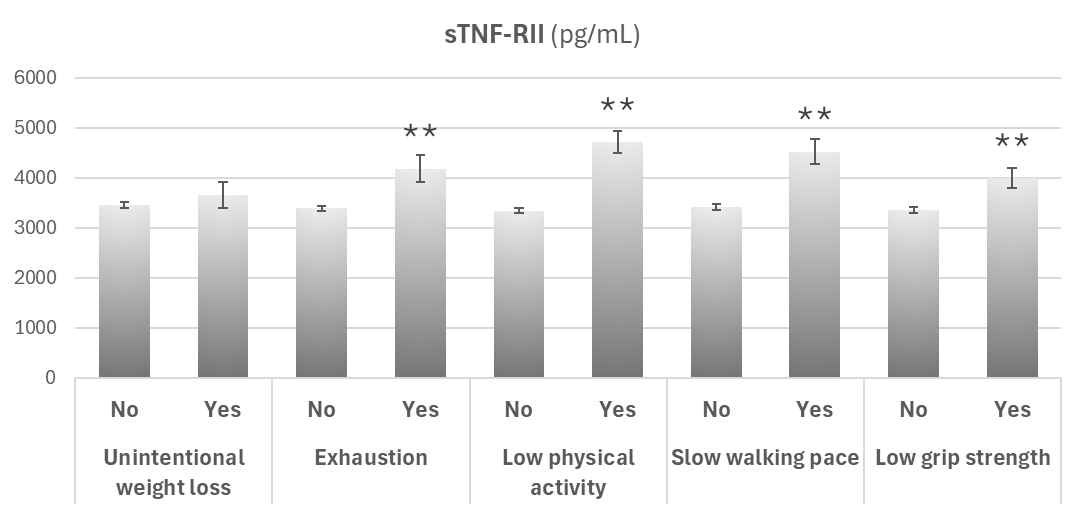

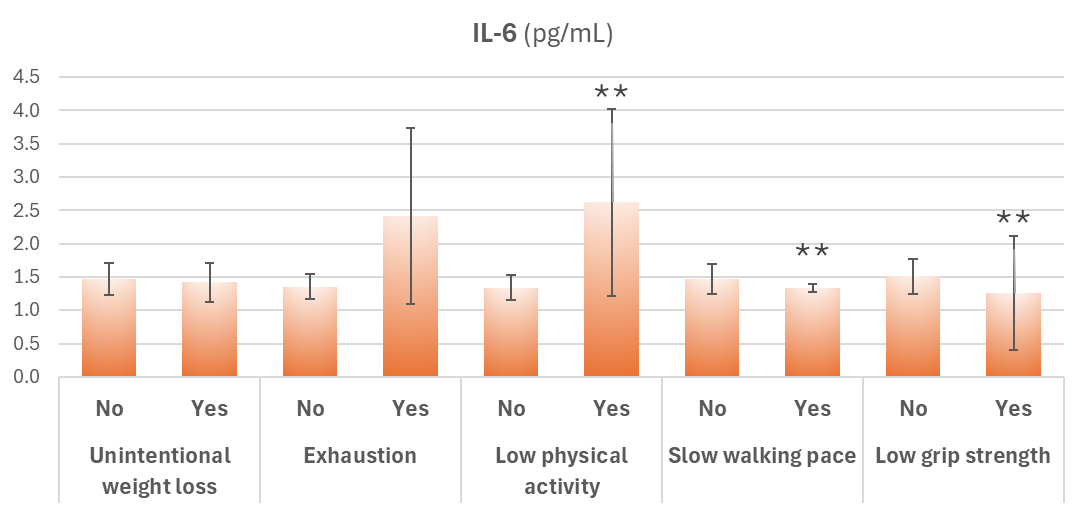

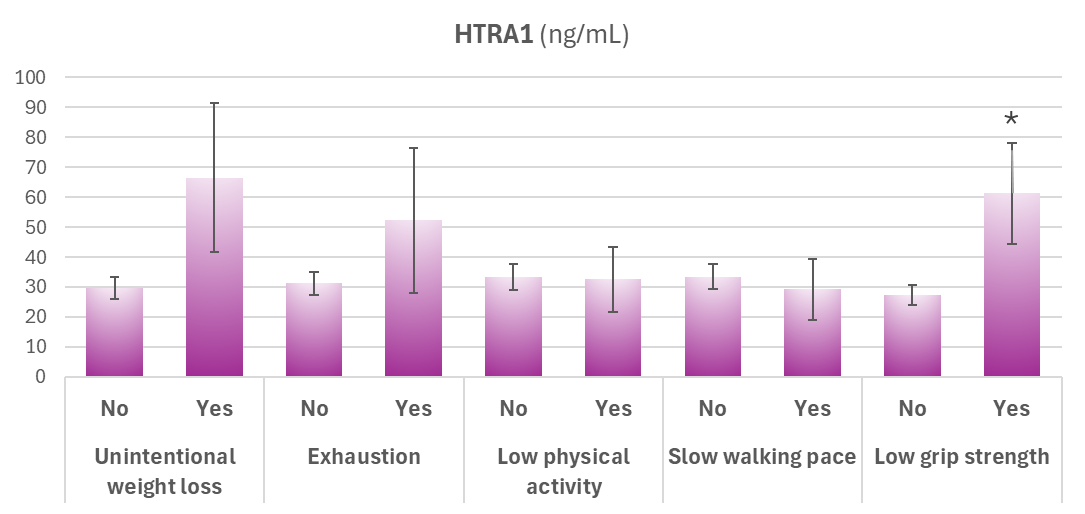

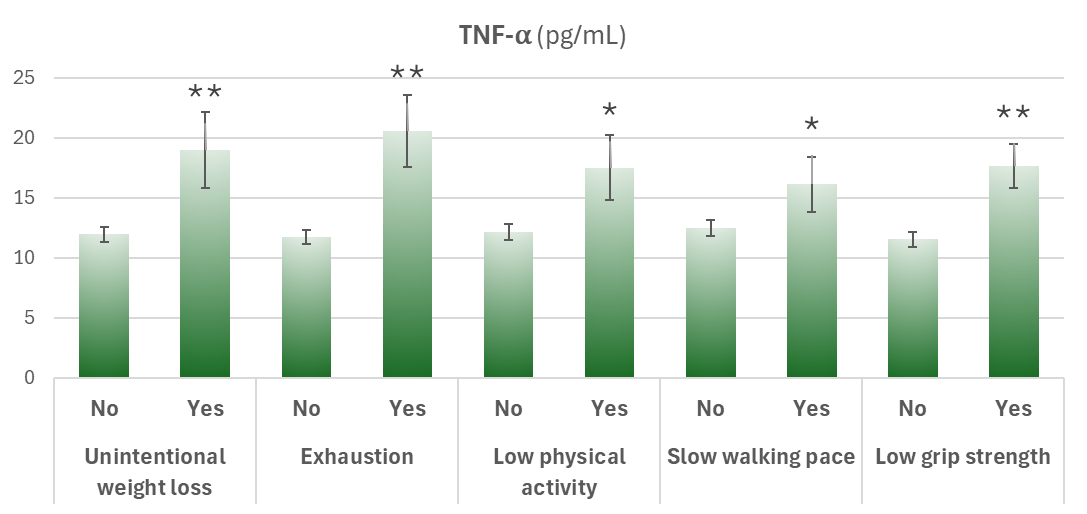

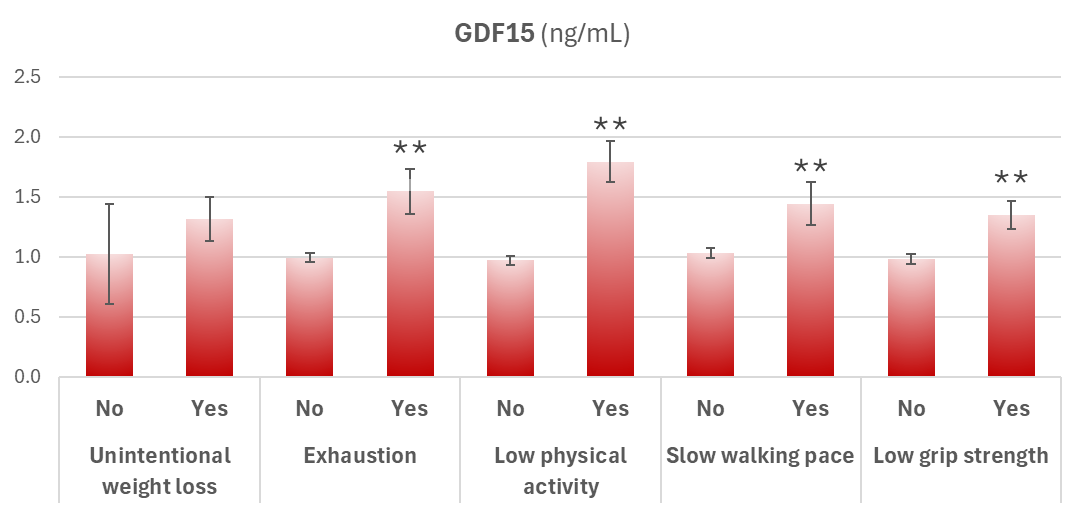

Supplement: Supplementary file 1 — Supplementary Material 1 (DOCX 1.02 MB) [file 11357_2025_1931_MOESM1_ESM.docx]
